# A Tetra-PEG Hydrogel Based Aspirin Sustained Release System Exerts Beneficial Effects on Periodontal Ligament Stem Cells Mediated Bone Regeneration

**DOI:** 10.3389/fchem.2019.00682

**Published:** 2019-10-17

**Authors:** Yunfan Zhang, Ning Ding, Ting Zhang, Qiannan Sun, Bing Han, Tingting Yu

**Affiliations:** ^1^Department of Orthodontics, Peking University School and Hospital of Stomatology & National Engineering Laboratory for Digital and Material Technology of Stomatology & Beijing Key Laboratory of Digital Stomatology, Beijing, China; ^2^School of Life Science and Medicine, Dalian University of Technology, Panjin, China

**Keywords:** hydrogel, drug delivery system, periodontal ligament stem cell, bone regeneration, aspirin

## Abstract

Bone defects, massive bone defects in particular, is still an issue clinically. Acetylsalicylic acid (ASA), also known as aspirin, has been proven to be conducive for mesenchymal stem cells osteogenic differentiation, which may be benefited for bone regeneration. In order to achieve a more appealing prognosis of bone defect, here we develop a well-defined tetra-PEG hydrogel sealant with rapid gelation speed, strong tissue adhesion, and high mechanical strength. After *in-situ* encapsulation of aspirin, this drug-loaded tetra-PEG hydrogel possessed a sustained release, anti-inflammation, and osteoinductive properties. *In vitro* experiments showed that the cell proliferation was slightly facilitated, and the osteogenic differentiation was notably augmented when periodontal ligament stem cells (PDLSCs) were co-incubating with the hydrogel materials. Moreover, *in vivo* study manifested that the aspirin sustained release system significantly facilitated the PDLSCs mediated bone defect regeneration. Overall, tetra-PEG hydrogel-based aspirin sustained release system is applicable not only for enhancing the osteogenesis capacity of PDLSC but also providing a new thought of bone regenerative therapy.

## Introduction

Autologous and allogenic bone grafts are currently the most common used therapeutic strategies for treating bone defect (Miller and Chiodo, 2016; Panagopoulos et al., 2017). However, the high cost of the bone harvesting procedures accompanied with donor site inflammation, pain, and hematomas limited its therapeutic usage. In comparison to currently available treatment modalities, mesenchymal stem cells (MSCs) based bone tissue engineering was indicated as an advantageous alternative therapeutic option for bone tissue regeneration (Botelho et al., 2017; Confalonieri et al., 2018), including high-quality regeneration capacity, low risk of autoimmune rejection, and no donor-site harvesting procedure.

Mesenchymal stem cells are existing in multiple tissue types, including the craniofacial, and dental tissues. It has been reported that orofacial tissues derived MSCs obtained superior proliferation, immunomodulation, and multiple-lineage differentiation abilities when compared with bone marrow derived mesenchymal stem cells (BMMSCs) (Gronthos et al., 2000; Seo et al., 2004; Zhang et al., 2009). Moreover, the neural crest origin of these MSCs makes them attractive for craniofacial regeneration strategies (Zheng et al., 2009; Xuan et al., 2018). Among the dental derived MSCs, periodontal ligament stem cells (PDLSCs) is of particular interest. It was reported that *ex vivo*-expanded PDLSCs was capable of achieving better bone regeneration capacity compared with other types of dental derived MSCs (Moshaverinia et al., 2014), and they could be easily collected in dental clinic from discarded tissue samples.

Mesenchymal stem cells biological behaviors can be affected by various factors, the recipient local microenvironment in which immune cells and cytokines may modulate MSCs mediated bone regeneration capacity (Liu et al., 2011). Acetylsalicylic acid (ASA) is one of the most widely used non-steroidal anti-inflammatory drugs (NSAIDs) that affects multiple biological process. It has been reported that ASA could be used in rodent osteoporosis treatment through activated osteoblasts and inhibited osteoclasts (Yamaza et al., 2008). When treated on MSCs, ASA is capable of elevating BMMSCs-mediated bone regeneration (Liu et al., 2011) and improving stem cells from human exfoliated deciduous teeth (SHED) osteogenic differentiation capacity (Liu et al., 2015). However, as ASA possessed a rapid dissolution profile and short half-life *in vivo* (Bliden et al., 2016), fabricating a suitable scaffold and delivery system to carry and sustain ASA efficacy at the site of bone repair is essential.

Among the biomaterials, hydrogels possess great potential in utilizing as delivery scaffolds for bone regeneration (Tan et al., 2019; Xu et al., 2019). Unlike other sustained release drug delivery systems, nanoparticles, for instance, hydrogels are comprised of a large amount of water within their 3D networks, which are excellent biomimicry for extracellular matrix. As tissue engineering scaffolds, hydrogels compose variable molecules which endue hydrogels diverse mechanical and biological properties (Seliktar, 2012), which have attracted great attentions in applications of drug release matrices, tissue-engineering scaffolds and coating biomaterials. However, most of hydrogels are generally mechanically soft or brittle, significantly limiting their scope of applications. A series of high-tough hydrogels like nanocomposite (NC) hydrogels, sliding-ring (SR) hydrogels, tetra-polyethylene glycol hydrogels (tetra-PEG hydrogels), ionically cross-linked hydrogels, and double-network (DN) hydrogels were well-developed in recent years (Tao et al., 2009; Yang et al., 2016, 2018; Bu et al., 2017). Wherein, tetra-PEG hydrogels were recognized as an ideal homogeneous biomaterial on account of the essentially non-immunogenic, antifouling, and biocompatible properties. In addition, tetra-PEG hydrogels have more advantages on facilely functional modification for construction of more-functional biomaterials in a convenient and practical way. In the present study, we investigated whether tetra-PEG hydrogels loaded with aspirin (PEG-ASA) complex is a suitable scaffold for delivering aspirin locally, and we hypothesized that the PEG-ASA complex might serve as an ideal approach for PDLSCs-mediated bone regeneration. We established the critical sized cranial bone defect on mice and analyzed the capability of the PEG-ASA complex to promote PDLSCs-mediated bone repair. The data may provide a new therapeutic strategy for achieving anti-inflammation and bone regeneration in repairing cranial bone defects.

## Materials and Methods

### Human PDLSCs Isolation and Cultivation

Periodontal ligament tissues were acquired from healthy premolars due to orthodontic treatment. The donors were aged from 18 to 25 years without any history of periodontitis or tooth decay. The protocol of PDLSCs isolation and cultivation was in accordance with previous publication (Seo et al., 2004). P3 cells are used in all experiments. The experiment procedure was approved by the Ethical Guidelines of Peking University (PKUSSIRB-201311103).

### *In vitro* Osteogenic Differentiation Assay

2 × 10^4^ PDLSCs were seeded per well in 12-well plates (Corning Incorporated, USA). The cells were cultured in growth medium (GM) containing α-modified Eagle's medium (Corning Incorporated), 15% fetal bovine serum (FBS, Biological Industries, Israel), and 1% penicillin/streptomycin (Solarbio Life Sciences, China) at 37°C and humidified 5% CO_2_. Then growth medium was replaced by osteogenic differentiation medium (ODM) containing α-modified Eagle's medium (Corning Incorporated), 15% FBS (Biological Industries), 1% penicillin/streptomycin (Solarbio Life Sciences), 0.01 μM Dexamethasone sodium phosphate (Sigma-Aldrich, USA), 1.8 mM KH_2_PO_4_, 0.1 mM L-ascorbic acid phosphate (Sigma-Aldrich), and 2 mM glutamine (Gibco, USA) when the cell confluence reached 70–80%. The ASA (Cat. A2093, Sigma-Aldrich) and hydrogel degradation (HD) was added into medium at the same time to reach a specific concentration (ASA: 0, 50, 100, 200, and 400 μg/mL; HD: 0 μg/mL, 10 μg/mL). The medium was replaced every 2 days. Alizarin red s staining was conducted at day 14 after osteogenesis induction. The cells were fixed by 60% isopropanol. After rehydrated in distilled water, 1% Alizarin red s (Sigma-Aldrich) solution was used to stain. The stain was removed. Cells were rinsed by distilled water 3 times and dried at room temperature. ImageJ (ver. 1.8.0; NIH, USA) were used to quantify the stained areas and shown as a percentage of the total area.

### Real-Time PCR

PDLSCs were cultivated in ODM with specific concentration of ASA (0, 50, 100, 200, and 400 μg/mL) and HD (0 μg/mL, 10 μg/mL) for 7 days. The total RNA was extracted following manufacturer's instruction by TRIzol reagent (Cat. 15596026, Invitrogen, USA). RNA concentration was measured by NanoDrop 8000 (Thermal Fisher Scientific, USA). 1 μg total RNA was reverse-transcribed to cDNA by PrimeScript RT Reagent Kit (Cat. RR037A, Takara Bio Inc., Japan). Quantitative PCR (qPCR) was performed by FastStart Universal SYBR Green Master (Cat. 04913914001, Roche, Swiss) on ABI Prism 7500 Real-Time PCR System (Applied Biosystem, USA). The gene expression was normalized by *GAPDH*. The result was analyzed by $2^{\left(-\Delta \Delta C_{\text{T}}\right)}$ method. The primers sequences are listed in Table 1.

**Table 1:** Sequences of quantitative polymerase chain reaction primers.

**Gene**	**Forward primer (5^**′**^-3^**′**^)**	**Reverse primer (5^**′**^-3^**′**^)**
*GAPDH*	GGAGCGAGATCCCTCCAAAAT	GGCTGTTGTCATACTTCTCATGG
*RUNX2*	TGGTTACTGTCATGGCGGGTA	TCTCAGATCGTTGAACCTTGCTA
*ALP*	AACATCAGGGACATTGACGTG	GTATCTCGGTTTGAAGCTCTTCC
*OCN*	CACTCCTCGCCCTATTGGC	CCCTCCTGCTTGGACACAAAG

*GAPDH, glyceraldehyde-3-phosphate dehydrogenase; RUNX2, runt-related transcription factor 2; ALP, alkaline phosphatase; OCN, osteocalcin*.

### Synthesis of Tetra-PEG-SG

The tetra-PEG-SG was prepared in two steps. Firstly, the intermediate product tetra-armed poly (ethylene glycol) glutarate acid was prepared as follows: tetra-PEG-OH (0.1 mmol, 10 g), glutaric anhydride (4 mmol, 456 mg), and DMAP (4 mmol, 488 mg) were dissolved in anhydrous CH_2_Cl_2_ (150 mL). After reaction for 24 h, the solution was washed with brine for three times. The organic layer was collected, dried with MgSO_4_, and concentrated under vacuum, which was further precipitated twice in excess diethyl ether to give the tetra-armed poly (ethylene glycol) succinic acid. Then the tetra-armed poly (ethylene glycol) succinic acid (0.05 mmol, 5 g), EDCI (2 mmol, 384 mg), and NHS (2 mmol, 230 mg) were dissolved in dry CH_2_Cl_2_ (100 mL). The system was stirred at 37°C for 24 h and then directly washed with brine (3 × 100 mL). The organic layer was collected and dried with MgSO_4_ to obtain the white solid under vacuum. The structures of the compounds were confirmed by ^1^H NMR and ^13^C NMR measurement in CDCl_3_. Yield: 62.5%.

### Preparing of Tetra-PEG Hydrogel and ASA-Loaded Tetra-PEG Hydrogel

Precursor solution of tetra-PEG-NH_2_ (8 wt%) and precursor solution tetra-PEG-SG (8 wt%) were prepared in two different sample bottles. By simultaneously injecting them into the molds using dual syringe, the tetra-PEG hydrogel was obtained at room temperature within 1 min. The ASA-loaded hydrogel was obtained by mixing the tetra-PEG-NH_2_ (8 wt%) and tetra-PEG-SG (8 wt%) including the appropriate amount of ASA (100 μg/mL for each experimental sample) into the molds at room temperature with the same methods.

### *In vitro* Release Profile of Aspirin From the Hydrogel

The hydrogel was prepared in a container with the diameter of 10 mm and height of 2 mm, and aspirin was encapsulated inside the hydrogel. Then, the ASA-contained hydrogel was immersed into the PBS and the solutions were collected at the appointed intervals of time. The collected solution at different time were tested using the UV-visible spectroscopy.

### Scanning Electron Microscopy (SEM) Observation of the Hydrogel

The hydrogel samples(φ 14 mm × h 2 mm) were lyophilized by SPEX 6770 freeze drier (Labconco, USA). To characterize the internal microstructure of hydrogels, the freeze-dried samples were cut and observed under a scanning electron microscopy (SEM, Hitachi S-4800, Japan).

### Cell Viability and Cell Proliferation Assay

Cell Counting Kit-8 (CCK-8, Cat. CK04, Dojindo, Japan) was applied under manufacturer's protocol. PDLSCs were seeded in 48-well plate (Corning Incorporated), 6,000 cells per well. GM was replaced, HD was added 24 h after seeding. Briefly, the cells were incubated with CCK-8 for 2 h, and OD value (450 nm) was detected. 24 h cell viability was defined as OD ratio between treated and untreated groups.

Cell live and dead viability was determined by Live/Dead Viability/Cytotoxicity Kit (Cat. L3224, Invitrogen) according to the manufacturer's instruction. PDLSCs were seeded and incubated in GM for 24 hours, HD was added and incubating for further 24 hours. IX53 fluorescence microscope (Olympus, Japan) was used to observe green (live) and red (dead) cells after staining.

PDLSCs were seeded on φ 20 mm slides (NEST, China), 1 × 10^4^ cells per slide. Cell treatments were the same as live and dead assay above. Then, PDLSCs were treated 12 h with 1:200 BrdU antibody (Cat. MA3-071, Invitrogen) after incubating the cells with 1:100 BrdU labeling reagent (Cat. 000103, Thermal Fisher Scientific) for 12 h. Then, irrigated cells were treated by Alexafluoro 568 conjugated secondary antibody for 1 h at room temperature. Finally, the slides were mounted by Vectasheild mounting medium containing DAPI (Vector Laboratories, USA). Zeiss Axio Observer Z1 (Zeiss, Germany) was used to examine the BrdU-positive cells.

### Toxicity Assay of Hydrogels *in vivo*

All the *in vivo* studies were approved by Peking University Biomedical Ethics Committee (LA2019074). Aged 8 weeks, female BALB/c nude mice were purchased from Weitonglihua (China). The nude mice were randomly divided into three groups: (1) control group; (2) tetra-PEG hydrogel group; (3) tetra-PEG hydrogel loaded with ASA group (for each group, *n* = 6), the materials were placed subcutaneously on the dorsum of the mice. The mice were euthanized 2 weeks postoperatively, the skin and subcutaneous implants were fixed by 4% neutral-buffered formaldehyde immediately after harvest for 2 days. All the samples were gradually dehydrated and embedded, sections (3 μm) were prepared and stained with hematoxylin and eosin (H&E) to evaluate the toxicity of the hydrogels in nude mice.

### Mouse Calvaria Bone Defects and Transplantation Treatment

Hydrogel samples (φ 5 × h 2 mm) were incubated in GM with PDLSCs for 48 h at 37°C, 5% CO_2_. The hydrogels were rinsed by PBS before implantation. Female C57BL/6 mice aged 8 weeks were purchased from Weitonglihua (China). Human PDLSCs used in this experiment were cultivated from one donor sample. Φ 5 mm calvaria bone defect was made by stainless-steel trephine. The mice are blindly randomized into 3 groups with the following: (1) control group, defects were not filled; (2) hydrogel-PDLSCs group, defect areas were filled by pretreated hydrogel matrix; (3) hydrogel-ASA-PDLSCs group, defect areas were filled by pretreated hydrogel with ASA loaded on. Then the mice were sacrificed at 8 weeks after operation. The calvaria bone was isolated and fixed with 4% neutral-buffered formaldehyde for 2 days. Then, the specimens were decalcified, dehydrated, and embedded. Serial sections 4 μm thick were prepared and stained with H&E and Masson to assess new bone formation. Local interferon-γ (IFN-γ) was assessed by immunofluorescence. IFN-γ antibody (Cat. sc-373727, Santa Cruz Biotechnology, USA) and goat-anti-mouse IgG-FITC (Cat. ZF-0312, ZSGB bio, China) was used in this experiment. Laser confocal microscopy (LMS710, Zeiss) was used to observe green and blue fluorescence emitted by FITC and DAPI.

### Micro Computed Tomography (Micro-CT) Analysis

Mice calvarial samples were radiographed with a micro-CT system (SkyScan 1174; Burker) at 53 kV and 810 μA. 3D image were reconstructed by using NRecon and CTvox software (Burker). The CTAn (Burker) software was used to analysis the volume of new bone.

### Statistics Analysis

All the results are presented as mean and standard deviation (mean ± S.D.) of 3–6 independent experiments. The statistics were analyzed by SPSS software (ver. 13.0; SPSS Inc., USA). Independent unpaired two-tailed Student's *t*-tests were used for comparing between two groups. One-way ANOVA was applied for more than two groups. *p* < 0.05 was considered to be significant.

## Result

### ASA Promotes the Osteogenic Differentiation of PDLSCs *in vitro*

**Figure 1:**
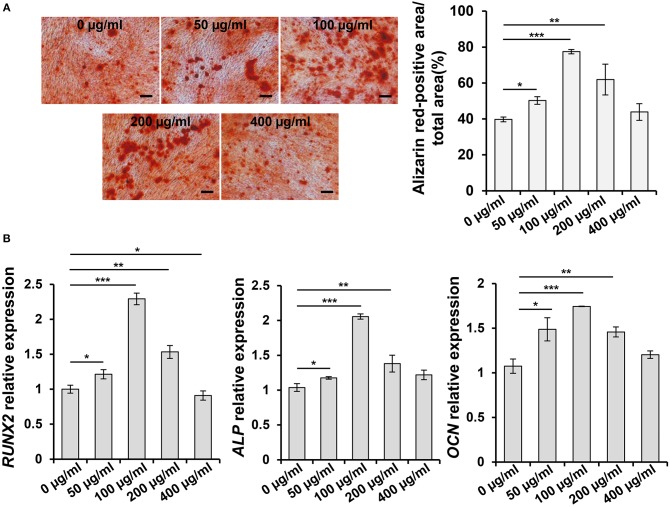
Aspirin promotes the osteogenic differentiation capacity of PDLSCs *in vitro*. **(A)** PDLSCs were treated with gradient concentration of aspirin (0, 50, 100, 200, and 400 μg/ml) for 14 days, the mineralized nodule formation was assessed by alizarin red staining. Scale bar, 200 μm. ^*^p < 0.05, ^**^p < 0.01, ^***^p < 0.001. **(B)** qPCR analysis showed the *RUNX2, ALP*, and *OCN* expression levels after 7 days gradient concentration of aspirin treatment under osteogenic induction condition. ^*^p < 0.05, ^**^p < 0.01, ^***^p < 0.001.

In order to investigate the effect of ASA on osteogenic differentiation of PDLSCs and select an appropriate concentration to encapsulate ASA in the tetra-PEG hydrogel, we set up a series doses of ASA to treat PDLSCs and analyzed its osteogenic potential. ARS staining was performed 14 days post-treatment. The result demonstrated that osteogenic differentiation of PDLSCs was enhanced with the increase of ASA from 0 to 100 μg/mL. However, the augmentation was attenuated with ASA concentration reached 200 and 400 μg/mL, in a dose-dependent manner. The measurement of mineralization areas approved that mineralized nodule formation capacity of PDLSCs peaked when treated with 100 μg/mL ASA (Figure 1A). Next, to further confirm the functional effect of ASA, the mRNA levels of osteogenic markers were evaluated 7 days after osteogenic induction as assessed by qPCR. The result demonstrated that there was a significant increase of runt-related transcription factor 2 (*RUNX2*), alkaline phosphatase (*ALP*), and Osteocalcin (*OCN*) at the dose of 100 μg/mL treated group when compared with control group (*p* < 0.001, Figure 1B). The mRNA expression profiles were consistent with those obtained from ARS staining. These data suggested that ASA is able to promote PDLSCs' osteogenic differentiation capacity, and 100 μg/mL might be an ideal concentration for ASA to be encapsulated during scaffold construction.

### Synthesis and Characterization of Hydrogels Loaded With ASA

**Figure 2:**
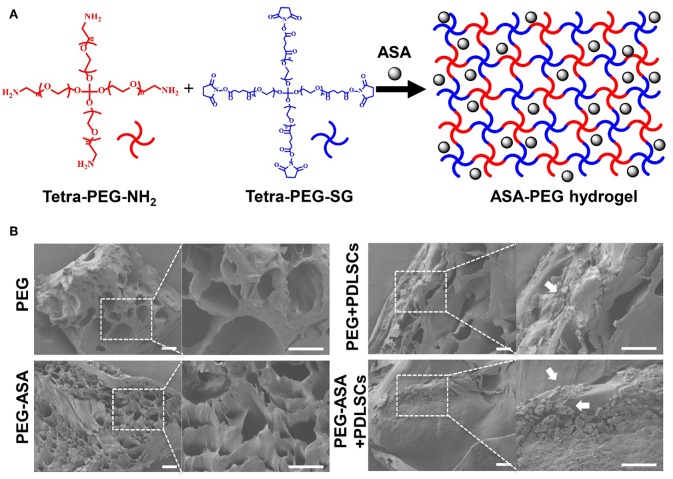
Scheme illustration and characterization of PEG hydrogels. **(A)** Scheme of the synthesis procedure for ASA-PEG hydrogels. **(B)** SEM images of PEG, PEG-ASA, and hydrogels co-cultured with PDLSCs. White arrowheads pointed out the cells. Scale bar, 100 μm.

Hydrogel and hydrogel loaded with ASA were fabricated with good accessibility by simply mixing the tetra-PEG-NH_2_ solution and the tetra-PEG-SG containing ASA solution (100 μg/mL) at room temperature. The synthesis procedures for PEG hydrogels were shown in Figure 2A. By the use of a dual syringe method, the tetra-PEG hydrogels can be formed in any mold with desired shape, exhibiting the injectable materials of *in-situ* free-shapeable properties. The topography and roughness of the hydrogels were investigated by the SEM technique as observed in Figure 2B. SEM images showed a varied of inner porous microstructure of the tetra-PEG hydrogels. It reveals that the pores diameters were ranging from 40 to 80 μm in the lyophilized state, which provided good chance to enable the sustained release of the encapsulated ASA drugs, cell entry and substance exchange intra-extra of the tetra-PEG hydrogels. As our expectation, we encapsulated ASA in PEG hydrogel without changing its appearance characteristics. Images of PEG and PEG-ASA co-cultured with PDLSCs manifested the excellent cell adhesion capacity of the hydrogels.

**Figure 3:**
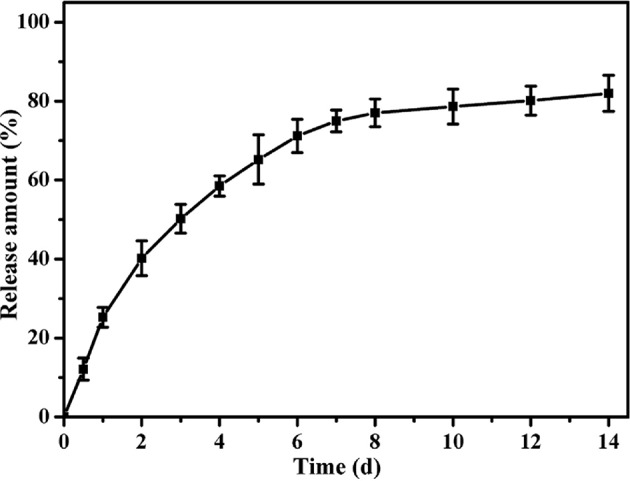
The releasing curves of aspirin from PEG hydrogels.

After the characterizations of hydrogels, we detected the release profile of ASA loaded in tetra-PEG hydrogel *in vitro*. As shown in Figure 3, constant and sustained release of ASA was observed up to 14 days. In the first 2 days, cumulative release of ASA quickly reached round 40%. This initial burst release of ASA could afford sufficient stimuli to the defect areas. Then the release rates of ASA approached its plateaus at day 8, and the cumulative release rate of ASA reached 80% at day 14. This result indicated a sustained release profile of ASA loaded within tetra-PEG based hydrogel.

### Cell Viability and Proliferation

**Figure 4:**
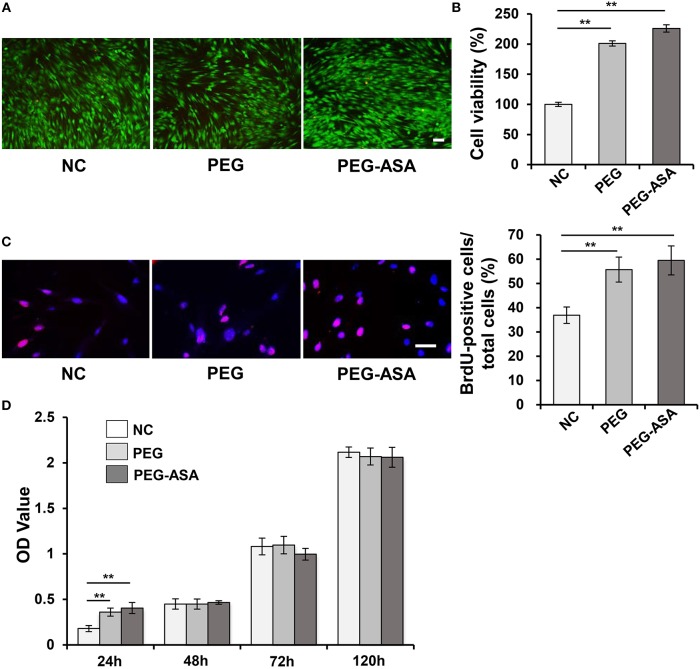
Cytotoxicity of hydrogels *in vitro*. **(A)** Live/dead staining of PDLSCs. Cells in green manifest living PDLSCs, while cells in red manifest dead ones. Scale bar, 100 μm. **(B)** Cell viability was detected by Cell Counting Kit-8 after 24 h cultivation. ^**^p < 0.01. **(C)** The proliferating PDLSCs was labeled in red and the percentage of Brdu-positive cells was calculated. Scale bar, 200 μm. ^**^p < 0.01. **(D)** Cell proliferation was detected at specific time points (24, 48, 72, 120 h) by using CCK-8. ^**^p < 0.01. NC, negative control.

As we have manufactured a feasible tetra-PEG based hydrogel scaffold loaded with ASA. In order to investigate its biocompatibility, series of cell proliferation, and cytotoxicity assays were set up. Live/dead assay confirmed that in the early stages of the culture (24 h), as it demonstrated in Figure 4A, PDLSCs manifest high viability. Quantitatively, PEG-ASA and PEG not only maintained but also improved cell viability vs. control group at 24 h (*p* < 0.01, Figure 4B). These results demonstrated hydrogels exerted low cytotoxicity on PDLSCs. To investigate if PEG-ASA and PEG are able to support the proliferation of PDLSCs. The BrdU assay was carried out, the result showed that the percentage of BrdU positive cells were elevated significantly in group PEG-ASA and group PEG after 24 h incubation (*p* < 0.01, Figure 4C). In addition, to observe a long-term proliferation profile on PDLSCs, CCK8 assay was conducted and OD value at 450 nm was measured at 48, 72, and 120 h after treatment with PEG and PEG-ASA. Interestingly, no significant difference between groups at these time point (Figure 4D) was found. These results indicated that the cell proliferation rate was promoted at early phase (24 h) of co-culturing with PEG and PEG-ASA, and it was not significantly inhibited at later phase (48, 72, and 120 h).

### PEG-ASA Promote Osteogenic Differentiation of PDLSCs *in vitro*

**Figure 5:**
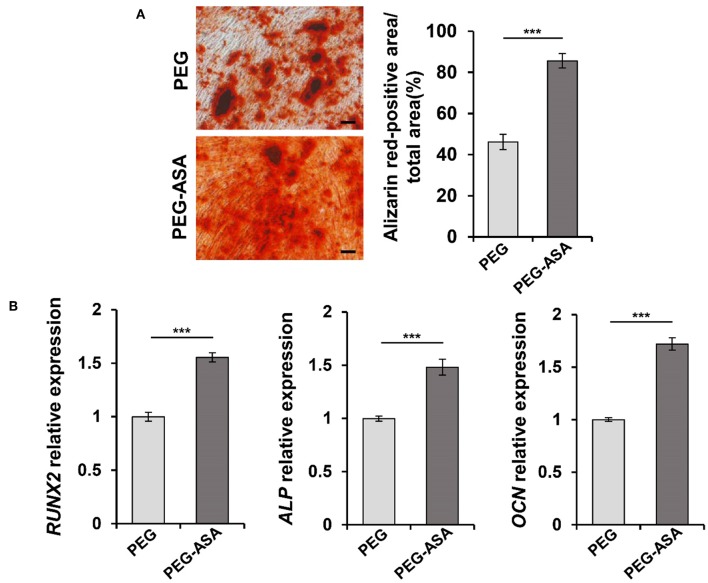
PEG-ASA promote osteogenic differentiation of PDLSCs *in vitro*. **(A)** PDLSCs incubated with hydrogels degradation for 14 days, the mineralized nodule formation was assessed by alizarin red s staining. Scale bar, 200 μm. ^***^p < 0.001. **(B)** qPCR analysis showed the *RUNX2, ALP*, and *OCN* expression levels after 7 days hydrogels degradation treatment. ^***^p < 0.001.

In the previous studies, we manifested that PEG-ASA possessed a low cytotoxicity characteristic and was able to sustain the proliferation of PDLSCs. Next, to investigate its osteoinduction ability, ARS staining and qPCR assay were used to examine. As shown in Figure 5A, the result indicated that PEG-ASA significantly increased the calcification nodules formation ability of PDLSCs after 14 days osteogenic induction. The expression levels of osteogenesis markers *RUNX2, ALP*, and *OCN* was significantly leveled up at day 7 in ASA sustained release system (*p* < 0.001, Figure 5B). These data verified our hypothesis that PEG-ASA exhibited osteoinductive ability on PDLSCs *in vitro*.

### Toxicity of PEG-ASA Hydrogels *in vivo*

**Figure 6:**
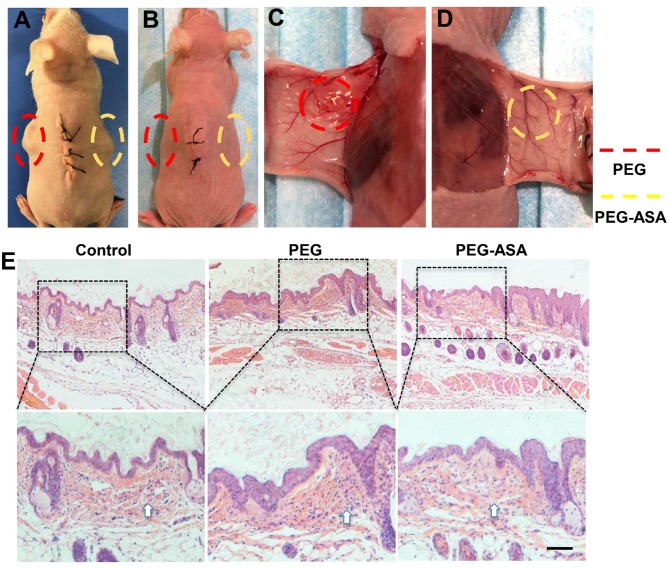
*In vivo* evaluation of the toxicity and degradation of the hydrogels. **(A)** Hydrogels cuboids were placed subcutaneously in BALB/c nude mice. The gross view **(B–D)** and subcutaneous tissue histology **(E)** of implant sites 2 weeks post-surgery. Scale bar, 200 μm. Implant sites of hydrogels were circled by dotted lines. The arrowhead indicated the inflammatory cells.

To validate the toxicity and degradation of hydrogels *in vivo*, subcutaneous implant assay on BALB/c nude mice was carried out (Figure 6A). Two weeks after surgery, both PEG and PEG-ASA hydrogels were almost absorbed *in vivo* from the general observation (Figure 6B). Macroscopically, the surrounding tissues of PEG groups displayed a slight inflammation response with mild local redness and swelling (Figure 6C). However, the surrounding tissues located around implanted PEG-ASA displayed no obvious inflammation response, with no significant redness, and exudate (Figure 6D). Since the nude mice lack of immune organs, BALB/c mice were used as an ideal animal model for the toxicity evaluation *in vivo*. The hydrogels along with surrounding tissues were collected and analyzed using H&E staining. From the histological examination, we observed that both PEG and PEG-ASA were biodegradable in 14 days, with no scaffold structures were found subcutaneously. Moreover, an increase amount of the inflammatory cells was detected located at epithelium tissues in PEG groups compared with no treatment control group (Figure 6E). The PEG-ASA displayed an inhibitory effect on aggregation of inflammatory cells which may attribute to the sustained release of aspirin. These results indicated that PEG hydrogel is a biodegradable scaffold and is not able to initiate severe inflammation response *in vivo*. PEG hydrogels loaded with or without ASA can be serve as a biocompatible scaffold for further therapeutic usage.

### PEG-ASA Improves PDLSCs-Mediated Bone Regeneration in Calvaria Bone Defect Model

**Figure 7:**
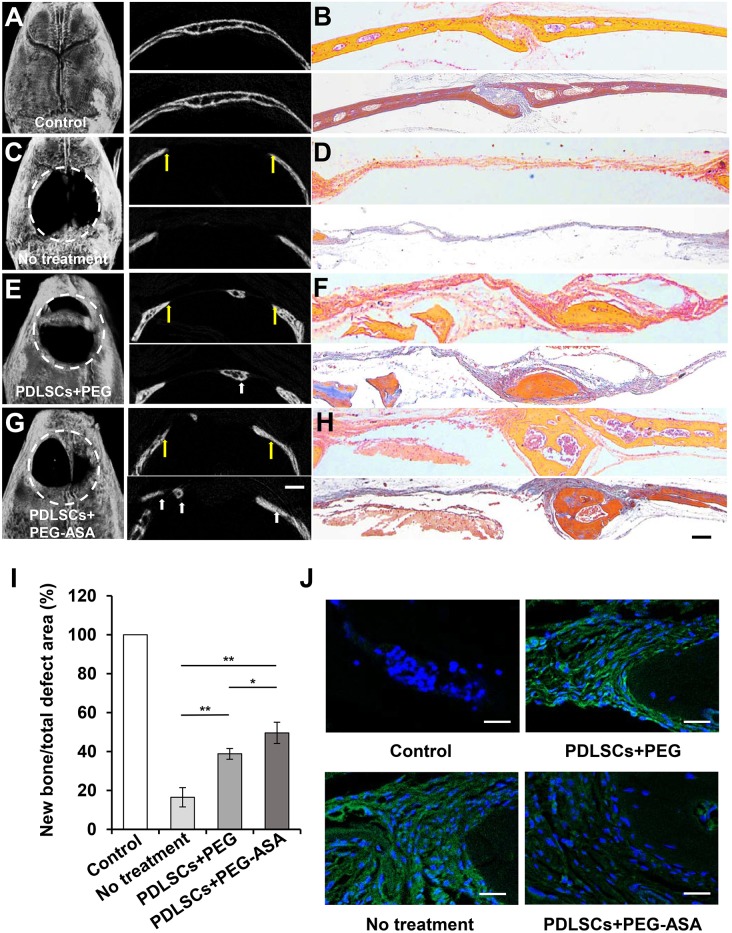
PEG-ASA improved PDLSCs-mediated critical-sized calvaria bone-defect bone formation. **(A)** Microcomputed tomography (micro-CT), **(B)** H&E (upper) and Masson staining (lower) images of unmanipulated wild-type C57BL/6 mice calvaria bone structure. **(C)** Micro-CT, **(D)** H&E (upper), and Masson staining (lower) images of untreated experimentally-induced calvaria bone defects. **(E)** Micro-CT (left), **(F)** H&E (upper), and Masson staining (lower) images of calvaria bone defects after transplantation of PDLSCs and PEG hydrogels. **(G)** Micro-CT (left), **(H)** H&E (upper), and Masson staining (lower) images of calvaria bone defects after transplantation of PDLSCs and PEG-ASA hydrogels. The yellow arrows in **(A,C,E,G)** indicate the margins of bone defects, the white arrows indicate the new bone. White dotted line defines the bone defect area. **(I)** Semiquantitative analysis of bone formation via micro-CT images from the groups described in **(A,C,E,G)**. **(J)** Immunofluorescence staining of IFN-γ positive cells. Scale bar, 1,000 μm (micro-CT), 200 μm (H&E and Masson), 25 μm (immunofluorescence). ^*^p < 0.05, ^**^p < 0.01.

According to the results above, PEG-ASA displayed a remarkable osteoinduction effect on PDLSCs *in vitro*. Thus, we hypothesized that the scaffold may also promote PDLSCs-mediated bone regeneration *in vivo*. Herein, we established the critical sized calvaria bone defect mice model and transplanted PDLSCs with hydrogels to the bone defect region. 8 weeks post-transplantation, Micro-CT images illustrated that PEG-ASA group obtained the newest bone regeneration than PEG alone group (Figures 7A,C,E,G). Statistical analysis showed that the area of new bone formation in the PEG and PEG-ASA group were much higher than no treatment control group. The PEG-ASA treated group displayed a significant elevation of new bone formation than the PEG group (Figure 7I).

To evaluate the amount of bone tissue regeneration, the histological section of the experimental region was made and stained by hematoxylin and eosin (H&E) and Masson's trichrome (Figures 7B,D,F,H). The PEG and PEG-ASA groups showed an increased PDLSCs-mediated bone formation compared to the untreated group, and PEG-ASA group displayed the most efficient treatment on mediating new bone formation. Immunofluorescence (Figure 7J) reveals relative low local inflammation status which might attribute to ASA laden. These results indicated that PEG-ASA could abate the inflammatory response during bone regeneration, and exerts beneficial effects on directing PDLSCs osteogenesis *in vivo*.

## Discussion

Massive bone defects, especially occurred in craniofacial area, make impacts on patients' aesthetics as well as function. Utilizing stem cells, biocompatible scaffolds and growth factors, tissue engineering provides more opportunities for bone tissue reconstruction. In this study, the viability and osteogenesis capability of PDLSCs cultured with tetra-PEG based hydrogel scaffold were measured for the first time. The tetra-PEG hydrogels established here is biocompatible, fully degradable, and capable of sustaining release of drug, which provided a promising scaffold for bone tissue engineering.

It has been reported that host proinflammatory T lymphocytes inhibit MSCs-mediated bone regeneration which induces cell apoptosis (Liu et al., 2011). Gibon et al. revealed that prolonged or aberrant immune activation is deleterious for bone regeneration (Gibon et al., 2017). These findings remained us that the host local immune response during calvaria bone defect repairment might play an important role in control MSCs-mediated bone tissue regeneration. Aspirin (ASA), widely used as analgesic and antipyretic for decades, is able to augment MSCs osteogenic potential through activate telomerase reverse transcriptase (Liu et al., 2015) (TERT) or inhibit tumor necrosis factor-α (TNF-α) and interferon-γ (IFN-γ) pathways (Liu et al., 2011). It is reported that MSCs osteogenesis was improved when exposed to relative low dosage (10–100 μg/mL) of ASA. Nevertheless, ASA shows significantly higher cytotoxicity and lower osteoinductivity when reached higher dosage (Cao et al., 2015; Liu et al., 2015; Yuan et al., 2018). In our study, we demonstrated that 100 μg/mL of ASA is the most efficient concentration for inducing PDLSCs toward osteogenic differentiation. Based on the beneficial effect of aspirin and our current findings, these evidences provide us the basis for fabricating a suitable delivery system for a sustained release of ASA to facilitate bone regeneration.

To achieve high-quality tissue regeneration, biomaterials have been used to control and manipulate the fate of stem cells (Engler et al., 2006; Fitzsimmons et al., 2018). In calvaria bone tissue engineering, the biomaterial plays an essential role to provide suitable microenvironment for supporting MSCs viability, proliferation, and directing the cells toward osteogenic differentiation (Moshaverinia et al., 2014). In our study, the viability of PDLSCs was significantly improved in both PEG and PEG-ASA hydrogels at 24 h, and the proliferation rate of PDLSCs was maintained as control group at 48, 72, and 120 h, which indicated the hydrogels we fabricated obtained a superior biocompatibility to support PDLSCs. Moreover, PEG hydrogel possesses a unique advantage as a drug carrier and contains almost no any harmful organics or crosslinking agents (Payyappilly et al., 2014). In our study, ASA loaded PEG hydrogels displayed a slowly release profile that could promote osteogenic differentiation of PDLSC both *in vivo* and *in vitro*. According to the previous reports, depending on the cell type and animal model, the most suitable concentration of ASA is between 50 and 200 μg/mL (Liu et al., 2011; Cao et al., 2015; Yuan et al., 2018). In our mice calvaria bone defect model, 100 μg/mL ASA were loaded on PEG hydrogels and located in the distinct bone defect area, the inflammation factors were inhibited along with the long-term release of ASA, which provide a suitable microenvironment for PDLSCs-mediated bone regeneration. Our result indicated that the area of new bone formation was largest with PEG-ASA hydrogel compared to PEG alone or the group with no treatment. These data suggest that PEG-ASA is safe and beneficial as a therapeutic method for clinic use.

## Conclusion

In this study, we fabricated a tetra-PEG hydrogel based aspirin sustained release system and demonstrated that the scaffold possesses an appropriate microenvironment for supporting PDLSCs' viability and proliferation. The *in vivo* study showed that both PEG and PEG-ASA hydrogels were capable of promoting PDLSCs-mediated calvaria bone regeneration in mice, and the effect of the PEG-ASA was more efficient than PEG alone. We anticipated that this finding may provide a new strategy for bone regenerative therapy.

## Data Availability Statement

The datasets generated for this study are available on request to the corresponding author.

## Ethics Statement

The studies involving human participants were reviewed and approved by The Ethical Guidelines of Peking University (PKUSSIRB-201311103). The patients/participants provided their written informed consent to participate in this study. The animal study was reviewed and approved by Peking University Biomedical Ethics Committee (LA2019074).

## Author Contributions

YZ and TY designed the experiments and wrote the manuscript. YZ, ND, TY, TZ, and QS performed the experiments and collected and analyzed the data. ND and BH designed the experiments and revised the manuscript. All co-authors approved the final version of the manuscript for publication.

### Conflict of Interest

The authors declare that the research was conducted in the absence of any commercial or financial relationships that could be construed as a potential conflict of interest.
